# Impact of Helicopter Emergency Medical Service in Traumatized Patients: Which Patient Benefits Most?

**DOI:** 10.1371/journal.pone.0146897

**Published:** 2016-01-15

**Authors:** Hagen Andruszkow, Uwe Schweigkofler, Rolf Lefering, Magnus Frey, Klemens Horst, Roman Pfeifer, Stefan Kurt Beckers, Hans-Christoph Pape, Frank Hildebrand

**Affiliations:** 1 Department of Orthopedic Trauma at Aachen University and Harald Tscherne Laboratory, University Hospital Aachen, Pauwelsstraße 30, 52074 Aachen, Germany; 2 Department of Trauma and Orthopedic Surgery, Berufsgenossenschaftliche Unfallklinik Frankfurt am Main, Friedberger Landstraße 430, 60389 Frankfurt am Main, Germany; 3 Institute for Research in Operative Medicine (IFOM), University of Witten/Herdecke, Ostmerheimer Str. 200, 51109 Cologne, Germany; 4 Emergency Medical Service Aachen, Department of Anaesthesiology, University Hospital Aachen, RWTH Aachen University, Pauwelsstraße 30, Germany; University of Michigan, UNITED STATES

## Abstract

**Introduction:**

The Helicopter Emergency Medical Service (HEMS) was established for the prehospital trauma care of patients. Improved rescue times and increased coverage areas are discussed as specific advantages of HEMS. We recently found evidence that HEMS exerts beneficial effects on outcomes for severely injured patients. However, it still remains unknown which group of trauma patients might benefit most from HEMS rescue. Consequently, the unique aim of this study was to reveal which patients might benefit most from HEMS rescue.

**Methods:**

Trauma patients (ISS ≥9) primarily treated by HEMS or ground emergency medical services (GEMS) between 2002 and 2012 were analysed using the TraumaRegister DGU. A multivariate regression analysis was used to reveal the survival benefit between different trauma populations.

**Results:**

The study included 52 281 trauma patients. Of these, 68.8% (35 974) were rescued by GEMS and 31.2% (16 307) by HEMS. HEMS patients were more severely injured compared to GEMS patients (ISS: HEMS 24.8±13.5 vs. GEMS 21.7±18.0) and more frequently suffered traumatic shock (SBP sys <90mmHg: HEMS 18.3% vs. GEMS 14.8%). However, logistic regression analysis revealed that HEMS rescues resulted in an overall survival benefit compared to GEMS (OR 0.81, 95% CI [0.75–0.87], *p*<0.001, Nagelkerke's R squared 0.526, area under the ROC curve 0.922, 95% CI [0.919–0.925]). Analysis of specific subgroups demonstrated that patients aged older than 55 years (OR 0.62, 95% CI [0.50–0.77]) had the highest survival benefit after HEMS treatment. Furthermore, HEMS rescue had the most significant impact after ‘low falls’ (OR 0.68, 95% CI [0.55–0.84]) and in the case of minor severity injuries (ISS 9–15) (OR 0.66, 95% CI [0.49–0.88]).

**Conclusions:**

In general, trauma patients benefit from HEMS rescue with in-hospital survival as the main outcome parameter. Focusing on special subgroups, middle aged and older patients, low-energy trauma, and minor severity injuries had the highest survival benefit when rescued by HEMS. Further studies are required to determine the potential reasons of this benefit.

## Introduction

Helicopter emergency medical service (HEMS) has been implemented in the preclinical treatment of trauma patients in diverse countries [1,2]. In some countries (e.g. Germany), HEMS rescue has been incorporated in a dense nationwide network of emergency medical services [1–5]. Nevertheless, the potentially beneficial effects of HEMS on patients' outcomes and cost efficiency are still controversial [1,3,6,7]. In this context, several disadvantages of HEMS such as the high financial burden [8] and the limited availability of HEMS, due to weather conditions or darkness have been reported [1,3,9]. However, presumable advantages compared to ground emergency medical services (GEMS) have also been described. In this context, HEMS is expected to facilitate rapid transport due to an increased transportation velocity [10]. Furthermore, HEMS medical crew members are supposed to be more experienced in trauma management, improving preclinical treatment of trauma patients [10,11] which might also result in improved triage and transportation to a specialist trauma centre thereby minimising inter-hospital transfers [12]. Also, the HEMS-related effects on posttraumatic mortality have been discussed controversially. While no significant effects of HEMS on mortality were found in some analyses [11,13,14], recent studies partly based on huge nationwide databases reported an independent benefit towards survival [1,6,15–18]. According to a current Cochrane Database analysis, these divergent results might be due to methodological issues, the considerable heterogeneity of health care systems (e.g. physician-staffed HEMS) and differences in the included study populations. In this context, it might be postulated that factors like demographic data and trauma mechanisms as well as injury severity or distribution might also affect the potential merits of HEMS rescue [7]. Two previous studies discussing these issues have been published using the German trauma registry [1,5]. As we have already demonstrated a significant benefit of HEMS rescue in Germany by these studies, the purpose of the current investigation was to examine whether age, gender, mode of injury, or injury severity could be used to determine specific trauma patient populations who might benefit explicitly from HEMS rescue.

## Materials and Methods

### The TraumaRegister DGU

The TraumaRegister DGU of the German Trauma Society (Deutsche Gesellschaft für Unfallchirurgie, DGU) was founded in 1993. The aim of this multi-centre database is anonymous and standardised documentation of severely injured patients.

Data are collected prospectively in four consecutive time phases from the site of the accident until discharge from hospital: A) Pre-hospital phase, B) Emergency room and initial surgery, C) Intensive care unit and D) Discharge. The documentation includes detailed information on demographics (age, gender), injury pattern, comorbidities, pre- and in-hospital management, course in the intensive care unit, relevant laboratory findings including data on transfusions and outcomes for each individual. The inclusion criteria are admission to hospital via emergency room with subsequent ICU/ICM care or reach the hospital with vital signs and die before admission to ICU. The infrastructure for documentation, data management, and data analysis is provided by AUC–Academy for Trauma Surgery (AUC–Akademie der Unfallchirurgie GmbH), a company affiliated to the German Trauma Society. The scientific leadership is provided by the Committee on Emergency Medicine, Intensive Care and Trauma Management (Sektion NIS) of the German Trauma Society. The participating hospitals submit their data anonymously into a central database via a web-based application. Scientific data analysis is approved according to a peer review procedure established by Sektion NIS. The participating hospitals are primarily located in Germany (90%), but a rising number of hospitals in other countries contribute data as well (at the moment from Austria, Belgium, China, Finland, Luxembourg, Slovenia, Switzerland, The Netherlands, and the United Arab Emirates). Currently, approximately 25 000 cases from more than 600 hospitals are entered into the database per year.

Participation in the TraumaRegister DGU is voluntary. For hospitals associated with TraumaNetzwerk DGU, however, the entry of at least a basic data set is obligatory for reasons of quality assurance. Actually, 58% of patients were treated in Level I trauma centres, 33% in Level II centres, and 9% in Level III centres.

This study followed the guidelines of the revised UN declaration of Helsinki in 1975 and its latest amendment in 1996 (42^nd^ general meeting). The study was approved by the internal review board of the Sektion NIS (Sektion Notfall, Intensivmedizin und Schwerverletztenversorgung) of the German Trauma Society (DGU). The present study is in line with the publication guidelines of the TraumaRegister DGU and registered as TR-DGU project ID 2013–033. Furthermore, this study was approved by the institutional ethical review board of the University Hospital Aachen, Pauwelsstraße 30, 52074 Aachen, Germany (No. EK 225/15). There was no need for written informed consent from the participants.

### Inclusion criteria

The present study included the following patients from the TR-DGU:

- Treated in a German Level I or II trauma centre
- Date of admission from January 2002 until December 2012
- Transportation to hospital either by physician staffed helicopter (HEMS) or physician staffed ground emergency medical services (GEMS)
- Primary admission from the scene of injury (inter-hospital transfers excluded)
- Early transfer out (<48h) excluded since final outcome not available
- Injury Severity Score (ISS) ≥ 9 points

### Injury severity and clinical outcome

The injury distribution was determined according to the Abbreviated Injury Scale (AIS, version 2005) and the overall injury severity was summarised by the Injury Severity Score (ISS) [19]. Furthermore, the New Injury Severity Score (NISS) was determined [20]. In order to assess the severity of traumatic brain injury (TBI), the first pre-hospital Glasgow Coma Scale (GCS) was used in addition to the AIS score [21]. Presence of a severe TBI was assumed if the AIS head score was ≥ 3 points and/ or GCS was ≤ 8 points.

The incidence of haemorrhagic shock in the preclinical setting was defined by a systolic blood pressure (SBP) ≤90mmHg.

The clinical course included the duration of mechanical ventilation as well as the length of intensive care unit and overall hospital stay [1]. Complications during hospital treatment included sepsis and organ failure [1]. The diagnosis of sepsis was made according to the criteria of the ACCP/SCCM consensus conference committee [22,23]. Organ function status was evaluated according to the Sequential Organ Failure Assessment (SOFA) score [24]. With three or more points an organ function was considered as failure while multiple organ dysfunction syndrome (MODS) was defined as simultaneous failure of at least two organs.

### On-scene treatment and mission times

Six different procedures of on-scene treatment were documented in the TR-DGU and analysed in order to evaluate potential differences in management between HEMS and GEMS. These procedures were intubation, insertion of a chest tube, application of vasopressors or sedatives, volume infusion, and cardio-pulmonary resuscitation (CPR). As HEMS and GEMS physicians are trained standardly in ATLS (Advanced Trauma Life Support) and PHTLS (Prehospital Trauma Life Support) in Germany, HEMS and GEMS physicians can be argued as comparably trained in these on-scene procedures and the prehospital management of trauma patients in general.

In addition, mission duration was defined as the time from accident until arrival at the emergency room.

### Statistics

Incidences are presented with counts and percentages while continuous values are presented as mean, standard deviation (*SD*) and median (*MD*). Due to the large sample size, even minor differences between the groups would become statistically significant, formal statistical testing was largely avoided. Selected differences were evaluated using the Chi-squared test for counts, and Mann-Whitney’s U-test for continuous variables. The interpretation of results, however, should focus on clinically relevant differences rather than on significant *p*-values.

Multivariate logistic regression analysis with in-hospital mortality as the dependent variable was performed in order to adjust for confounding variables. Besides the mode of transportation, the following variables were included as confounders in the statistical model: Time of treatment, ISS, age, gender, unconsciousness (GCS ≤ 8), AIS head (four and five points), shock (prehospital systolic blood pressure ≤ 90 mmHg), intubation, type of injury (blunt/ penetrating), mechanism of injury, level of care of the target hospital, and time of day (daytime/night). Results were reported as odds ratios with 95% confidence intervals (95% CI).

Subsequently, the logistic regression analysis was repeated in subgroups of patients using only the significant confounders identified in the first model as confounding independent predictor variables. From these subgroup analyses only the odds ratio (OR) of the HEMS (relative to GEMS) was reported, with 95% CI.

The data were analysed using the Statistical Package for the Social Sciences (SPSS; version 22; IBM Inc., Somers, NY, USA).

## Results

### Demographic data

**Fig 1:**
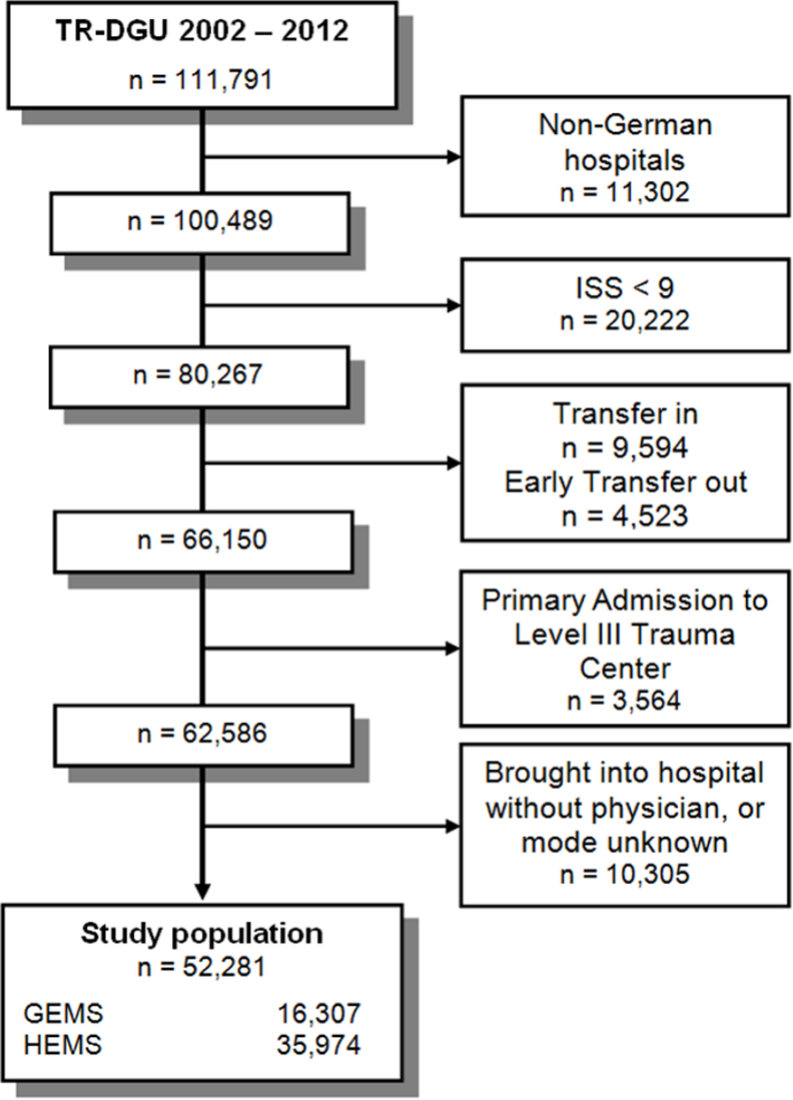
Study flow chart illustrating the selection of patients.

A total of 52 281 trauma patients were included in the present study (Fig 1). Of these, 68.8% (n = 35 974) were rescued by GEMS and 31.2% (n = 16 307) by HEMS. The mean age for all patients was 46.6 ± 21.4 years, and 72.2% were males. Patients transported by HEMS were younger (HEMS: 43.9 ± 20.3 years; GEMS: 47.8 ± 21.8) and more often of male gender (HEMS: 75.0%; GEMS: 70.9%). Overall, 69.3% (n = 36 212) of patients were transported to a Level I trauma centre, 30.7% (n = 16 069) to a Level II trauma centre.

### Cause of injury, injury distribution and on-scene treatment

Overall, 60.1% of included patients suffered from traffic accidents. HEMS patients suffered more often from high energy trauma, mainly road traffic accidents by car and motorcycle (Table 1). A total of 67.9% (n = 35 476) of all patients had an ISS ≥16 points. Patients rescued by HEMS sustained a higher injury severity (Table 2) associated with an increased incidence of on-scene traumatic shock (HEMS: 18.3% vs. GEMS: 14.8%).

**Table 1:** Trauma mechanism.

	All patients	HEMS	GEMS
Traffic—Car	13,811	33.4%	25.3%
Traffic—Motorbike	7,525	21.4%	12.3%
Traffic—Bicycle	4,053	7.5%	8.5%
Traffic—Pedestrian	4,000	4.4%	9.7%
High fall > 3m	8,828	16.8%	18.2%
Low fall < 3m	7,351	8.6%	17.6%
Others	4,064	7.9%	8.3%

**Table 2:** Demographics, injury distribution and injury severity.

	HEMS	GEMS
Age (years)	43.9 ± 20.3	47.8 ± 21.8
Gender (male)	75.0%	70.9%
Head	45.6%	41.7%
Chest	52.3%	45.2%
Abdomen	16.0%	13.5%
Extremities	37.8%	31.5%
ISS (mean ± SD) [median]	24.8 ± 13.5 [22.0]	21.7 ± 13.3 [18.0]
NISS (mean ± SD) [median]	30.5 ± 15.7 [27.0]	27.1 ± 14.9 [22.0]
ISS ≥ 16	74.5%	64.8%
24-hours mortality	7.6%	7.0%
Overall mortality	14.2%	13.3%

Patients treated by GEMS more often had isolated traumatic brain injuries (TBI) (HEMS: 9.6% vs. GEMS: 13.7%), while the incidence of TBI in severe trauma patients was higher in HEMS rescues (HEMS: 45.9% vs. GEMS: 40.0%).

Helicopter rescue teams performed more on-scene interventions (Table 3). Furthermore, a prolonged overall mission duration (HEMS: 76.6 ± 28.2 min. vs. GEMS: 60.7 ± 26.7 min.) and a longer on-scene time (HEMS: 37.0 ± 20.4 min. vs. GEMS: 28.0 ± 16.0 min.) compared to GEMS was observed with HEMS treatment.

**Table 3:** On-scene interventions.

On-scene management	HEMS	GEMS
Intubation	61.5%	31.6%
Chest tube insertion	9.0%	2.8%
Application of vasopressors	11.3%	7.3%
Application of sedatives	87.2%	71.5%
Volume infusion	95.6%	91.5%
Cardio-pulmonary resuscitation	3.0%	3.2%

### Posttraumatic complications, clinical treatment and outcomes

Evaluating the in-hospital clinical course, an increased incidence of multiple organ dysfunction syndrome (MODS) (HEMS: 30.1% vs. GEMS: 23.1%) and sepsis (HEMS 8.3% vs. GEMS 6.1%) was revealed for HEMS patients. In these patients, the duration of mechanical ventilation (HEMS: 6.4 ± 10.8 days vs. GEMS: 4.0 ± 8.7 days), ICU treatment (HEMS: 10.3 ± 13.2 days vs. GEMS: 7.5 ± 11.2 days) and overall length of stay (HEMS: 25.2 ± 26.6 days vs. GEMS: 19.6 ± 19.2 days) were also increased. Overall in-hospital mortality was 13.5% (n = 7 084), with 14.2% patients deceased after HEMS rescue and 13.3% after GEMS rescue.

### Outcome measurement and subgroup analysis

According to the logistic regression model, HEMS rescues resulted in a significant overall mean survival benefit compared to GEMS (OR 0.81, 95% CI [0.75–0.87], *p*<0.001), Nagelkerke's R squared 0.526, area under the ROC curve 0.922, 95% CI [0.919–0.925]). This result is adjusted for other independent predictors of survival such as injury severity, the presence of a severe TBI, a shock in the preclinical setting, penetrating trauma, and the level of care of the treating hospital (Table 4).

**Table 4:** HEMS rescue as independent survival predictor (n = 52,006). Only significant factors are presented.

Predictor towards mortality	Regression coefficient	Odds Ratio	95% CI	p-value
HEMS	-0.217	0.805	0.746–0.868	<0.001
Injury Severity (NISS, per point)	0.053	1.054	1.051–1.057	<0.001
Severe Traumatic Brain Injury (AIS ≥ 5)	0.864	2.372	2.159–2.606	<0.001
Penetrating trauma	0.375	1.455	1.240–1.707	<0.001
Shock (SBP ≤ 90mmHg)	0.740	2.097	1.933–2.274	<0.001
Hospital Level of Care (Level II)	0.112	1.118	1.034–1.209	0.005

The impact of HEMS in different subgroups, compared to the overall survival benefit of HEMS (OR 0.81), should identify trauma patients which benefit most from HEMS rescue in Germany. With respect to demographic aspects, elderly patients aged 55 years and above revealed the strongest survival benefit (Table 5). Regarding the trauma mechanism, HEMS rescue had the most benefit following ‘low falls’ while the outcome in traffic injuries was comparable with GEMS. With respect to the injury severity, major benefits were identified in patients with minor polytrauma (ISS less than 25) (Table 5).

**Table 5:** Multivariate analysis of HEMS versus GEMS in subgroups of patients. Instead of the full model only the OR for HEMS is reported which was 0.81 in the whole patient group (Table 4).

Subgroup	n	Odds Ratio for HEMS	95% CI
1–15 years	1,919	1.07	0.63–1.81
16–54 years	31,577	0.90	0.80–1.00
55–64 years	6,177	0.62	0.50–0.77
65–74 years	5,843	0.74	0.62–0.89
>74 years	6,490	0.72	0.61–0.85
Car accident	13,744	0.93	0.80–1.08
Motorcycle accident	7,489	0.95	0.74–1.20
Bicycle accident	4,030	1.08	0.83–1.42
Pedestrian accident	3,984	0.77	0.58–1.03
High fall	8,772	0.82	0.68–1.00
Low falls	7,317	0.68	0.55–0.84
ISS 9–15	16,732	0.66	0.49–0.88
ISS 16–24	15,950	0.73	0.61–0.88
ISS 25–33	10,208	0.78	0.68–0.89
ISS ≥34	9,116	0.90	0.80–1.01
Traumatic brain injury (AIS ≥3)	22,322	0.83	0.76–0.90
Chest trauma (AIS ≥3)	24,665	0.83	0.75–0.92
Abdomen trauma (AIS ≥3)	7,413	0.90	0.76–1.07
Pelvic trauma (AIS ≥3)	9,695	0.82	0.70–0.97
On-scene CPR	1,639	0.84	0.63–1.13

## Discussion

Since its introduction, the outcome benefit of HEMS rescue in trauma patients has been discussed controversially. Decisions for initiation of preclinical treatment and transportation of trauma patients by HEMS rescue are still not evidence based [7]. Furthermore, the significance of potential risks and benefits of HEMS treatment remain unclear [25]. The partly divergent results might be caused by the methodological weakness of some studies, the considerable heterogeneity of health care systems (e.g. physician-staffed HEMS), and different study methodologies [7]. Based on the beneficial effects of HEMS rescue observed in our previous study [1], we aimed to identify specific subgroups of trauma patients which might particularly benefit from HEMS rescue. This analysis was again based on one of the largest databases of trauma patients (TraumaRegister DGU). The main results might be summarised as follows:

1. In general, almost all subgroups of trauma patients benefit from HEMS rescue with in-hospital survival as the main outcome parameter (OR 0.81, 95% CI [0.75–0.87], *p*<0.001).
2. Patients treated by HEMS differed significantly in terms of age, trauma mechanism, injury distribution, injury severity, and on-scene trauma management compared to GEMS patients.
3. Elderly patients with low energy trauma had the greatest benefit from HEMS compared to GEMS.

In-hospital mortality has uniformly been suggested to represent the most important end-point outcome when analysing the impact of HEMS-associated preclinical care for multiple trauma patients [1,3,6,7,11–13]. In accordance with various other investigations based on large databases [6,16,26], we also found a beneficial effect of HEMS on posttraumatic mortality. This is in line with the results of Abe et al. who analysed the Japanese Trauma Databank [26]. The authors found an adjusted survival benefit of physician staffed HEMS with an odds ratio of 1.23. Furthermore, patients treated by helicopter differed from GEMS patients in terms of age, trauma mechanism, injury distribution and injury severity, and on-scene trauma management comparable to results of the present study. In 2010, Brown et al. analysed the National Trauma Databank comparing 41 987 HEMS and 216 400 GEMS patients [16]. The authors adjusted for injury severity, age, gender, injury mechanism, vital signs, level of trauma centre, and urgency of operation. Again, HEMS was associated with an improved survival similar to the effect reported in this study. The largest registry study with a study population of approximately 230 000 patients was currently performed by Galvagno et al. [6]. After adjustment for several confounding factors and a variance calculation to control for clustering by trauma centre, helicopter transport was associated with a lower mortality in Level I (OR 0.88) and Level II trauma centres (OR 0.86). Although this statistical aspect was argued to represent more valid data compared to previous studies [6], the comparison of differently trained rescue teams represents a potential limitation which has also previously been discussed [1,3,13,14]. In the present study, the presumable confounding factor of different rescue teams can be neglected due to the fact that exclusively physician staffed rescue teams were compared. However, other studies did not find a positive impact of HEMS transportation on outcomes in trauma patients [11,13,14,27]. In a current Cochrane Review, these divergent results led to the conclusion that the relevance of potentially beneficial effects of HEMS need further clarification. It has been suggested that the considerable heterogeneity of study populations and study methodologies in the current literature might be significant reasons for this diversity of study results [7].

We therefore performed an analysis of specific subgroups based on demographic data, injury mechanism as well as injury severity and distribution. Beside the overall benefit of HEMS rescue in almost all subgroups of trauma patients, we particularly found the most pronounced survival benefit in the group of elderly patients, in patients with low-energy trauma and in the group with a lower injury severity. This aspect demands a critical reflection due to the steadily increasing average age of trauma patients. As geriatric trauma patients have been identified as the fastest growing population in trauma centres with an estimation of 40% by the year 2050 [28–30], the challenge to prevent mortality in this trauma population will also involve the prehospital management in HEMS and GEMS rescue. Analysing potential trauma mechanisms in young and older patients, low-level falls as the major cause for low-energy trauma have been identified as the predominant cause of injury in patients aged over 65 years [30,31]. In this respect, Sterling et al. demonstrated that almost 50% of patients older than 65 years suffered from falls compared to only 7% in the younger generation [32]. Nevertheless, road traffic accidents represent the second most common mechanism of injury of elderly patients [33,34]. In accordance with the different trauma mechanisms between young and older patients, differences towards the treatment strategies were revealed [35,36]. Peschman et al. demonstrated that patients older than 65 years were more likely to be admitted to hospital compared to younger patients [35]. Despite this increased rate of hospital admission, older patients were more frequently triaged to non-trauma centres despite a proven favourable outcome in specialist trauma centres [31,33,36]. In this context, it was demonstrated that elderly trauma patients injured in traffic accidents had a lower mortality compared to those after low-energy trauma even when adjusted for injury severity and comorbidities [31,34]. This was explained by a longer delay in triage, and a longer time between the assessment and admission after falls when compared to traffic accidents [31,34]. Focusing on the presented survival rates in helicopter rescue in this study, the preferred admission to Level I trauma centres in HEMS rescue could be argued to be one potential reason for the improved survival in older patients. Furthermore, direct admission to hospital was guaranteed due to the inclusion criteria of this study.

Beside the impact of trauma care levels, further independent mortality risk factors in older trauma patients have been evaluated. Hranjek et al. found that the injury severity, blood pressure and mechanical ventilation were independent predictors of mortality in older trauma patients [37]. Even a minor injury severity between 16 and 19 ISS points had an odds ratio towards mortality of 2.4 in these patients [37]. Furthermore, hypotension has been observed in 20% of elderly trauma patients, and was associated with an increase of the mortality risk from 12% to 35% [36]. Consequently, an early and aggressive treatment strategy for older trauma patients has been noted by several studies [31,36,38]. In conclusion, the demonstrated improved survival in HEMS rescue might have been determined by the extended on-scene treatment by HEMS compared to GEMS and the direct hospital admission in this study.

In summary, elderly patients benefit from both, aggressive on-scene treatment and resuscitation as well as an early admission to a trauma centre with geriatric trauma experience regardless of the trauma mechanism. We suggest in this study that these aspects were guaranteed in HEMS rescue, influencing the impact of HEMS. Beside the HEMS rescue as a transportation platform, on-scene management and preferred admission to Level I trauma centres might have improved the survival in consequence.

The present study has limitations that need to be recognised. Although databank analyses include a large number of patients, their validity might be restricted due to detection of minor statistical differences without mandatory clinical relevance [1]. We therefore decided to present the descriptive variables of our study populations without statistical significances. Furthermore, we had to exclude approximately 9% of all patients due to missing data regarding the transportation mode. Although this might have influenced our results, we expect this bias to be of minor effect. In comparison, Galvagno excluded 40% due to missing disposition information [6]. Another bias could be expected by factors not evaluated by the databank (weather conditions, transportation distances, etc.). Further criticism could be mentioned regarding the inclusion criterion of an ISS ≥9 points. We decided to use the inclusion criterion of ISS ≥9 because many patients with an ISS between 9 and 15 were transported by helicopter. Little information is available regarding minor polytrauma treated by HEMS [39]. In addition, including patients with an ISS <16 has previously been established [1,40]. This study did not intend to evaluate the potential reason for the outcome benefit in HEMS rescue. Therefore, we did not analyse the influence of on-scene treatment procedures and mission times towards outcome although we provided these data in order to guarantee comparability to further studies in this field. In this respect, intubation especially as an on-scene procedure should be argued critically considering the reasons (Traumatic Brain Injury and unconsciousness, analgesia, etc.). Another important aspect should be considered. In general, patients under CPR are not transported by HEMS due to the small space that is available during HEMS transport. Patients transported by HEMS must have already returned to spontaneous circulation.

## Conclusions

In summary, trauma patients benefit from HEMS rescue with in-hospital survival as a main outcome parameter. Analysing different subgroups, older patients, low-energy trauma, and minor injury severity had the most pronounced survival benefit when rescued by HEMS. An aggressive on-scene treatment, a direct and early hospital admission, and preferred admission to Level I trauma centres might have influenced the survival in older patients in the case of HEMS rescue. Nevertheless, further studies are required to determine the potential reasons for this benefit.

## Abbreviations

AIS: Abbreviated Injury Scale
AUC: AUC—Akademie der Unfallchirurgie GmbH / AUC—Academy for Trauma Surgery
CI: Confidence interval
CPR: Cardiopulmonary resuscitation
DGU: German Trauma Society
GCS: Glasgow Coma Scale
GEMS: Ground emergency medical services
HEMS: Helicopter emergency medical services
ICU: Intensive Care Unit
ISS: Injury Severity Score
m: Meter (unit)
min.: Minute (unit)
MODS: Multiple Organ Dysfunction Syndrome
OR: Odds ratio
SBP: Systolic blood pressure
SD: Standard deviation
SOFA: Sequential Organ Failure Assessment
SPSS: Statistical Package for the Social Sciences
TR-DGU: TraumaRegister DGU
